# Protein Nanoparticles as Drug Delivery Carriers for Cancer Therapy

**DOI:** 10.1155/2014/180549

**Published:** 2014-03-20

**Authors:** Warangkana Lohcharoenkal, Liying Wang, Yi Charlie Chen, Yon Rojanasakul

**Affiliations:** ^1^Department of Pharmaceutical Sciences, West Virginia University, Morgantown, WV 26506, USA; ^2^Pathology and Physiology Research Branch, National Institute for Occupational Safety and Health, Morgantown, WV 26505, USA; ^3^Natural Science Division, Alderson-Broaddus College, Philippi, WV 26416, USA; ^4^Mary Babb Randolph Cancer Center, West Virginia University, Morgantown, WV 26506, USA

## Abstract

Nanoparticles have increasingly been used for a variety of applications, most notably for the delivery of therapeutic and diagnostic agents. A large number of nanoparticle drug delivery systems have been developed for cancer treatment and various materials have been explored as drug delivery agents to improve the therapeutic efficacy and safety of anticancer drugs. Natural biomolecules such as proteins are an attractive alternative to synthetic polymers which are commonly used in drug formulations because of their safety. In general, protein nanoparticles offer a number of advantages including biocompatibility and biodegradability. They can be prepared under mild conditions without the use of toxic chemicals or organic solvents. Moreover, due to their defined primary structure, protein-based nanoparticles offer various possibilities for surface modifications including covalent attachment of drugs and targeting ligands. In this paper, we review the most significant advancements in protein nanoparticle technology and their use in drug delivery arena. We then examine the various sources of protein materials that have been used successfully for the construction of protein nanoparticles as well as their methods of preparation. Finally, we discuss the applications of protein nanoparticles in cancer therapy.

## 1. Introduction

In the last decades, the growth of nanotechnology has opened several new possibilities in medical sciences, especially in the field of drug delivery. Different new drug carrier systems in the micro- and nanometer size range have been developed and the number of patents and products in the drug delivery field has increased tremendously [1]. Various nanotechnology platforms are being investigated in either the developmental or clinical stages in order to obtain more effective and safer therapeutics for a myriad of clinical applications. One of the most needed applications is in the area of cancer treatment in which several new products have been launched (Table 1). These nanoparticle drugs are poised to have a major impact on the treatment of oncologic diseases.

Nanoscale drug delivery systems that have been developed include liposomes and nanoparticles. Nanoparticles are solid colloidal particles ranging in size from about 10 nm to 1000 nm. The major goal in designing nanoparticles as a delivery system is to control particle size, surface properties, and release of pharmacologically active agents in order to achieve the site-specific action of drugs at a therapeutically optimal rate and dosage regimen [2, 3]. Nanoparticle delivery systems offer certain distinct advantages for drug delivery [4]. First, the particle size, particle morphology, and surface charge of nanoparticles can be controlled [5]. Secondly, nanoscale drug delivery systems can carry or deliver a variety of therapeutic and diagnostic agents such as small molecules (hydrophilic or hydrophobic), peptides, proteins, and nucleic acids while releasing the active molecules in a controlled manner. The entrapped molecules can be released from the nanocarriers in a precise manner over time to maintain drug concentrations within a therapeutic window, or they can be triggered to be released by some stimuli unique to the delivery site [6]. Thirdly, these nanocarriers can improve the solubility and stability of encapsulated drugs, providing an opportunity to reevaluate drug candidates that were previously ignored because of poor pharmacokinetics [7]. Lastly, site-specific drug delivery can be achieved using nanoparticles delivered through various routes of administration. The nanocarriers can be engineered to have a prolonged circulation time or to have enhanced cellular uptake and targeting abilities [8].

The development of nanoparticle-based drug delivery systems is rapidly growing due to their great therapeutic potential. Various types of materials including polymers, lipids, polysaccharides, and proteins have been explored as drug delivery carriers. The selection of nanoparticle materials is dependent on many factors including (a) the size of nanoparticles needed, (b) inherent properties of the drug such as aqueous solubility and stability, (c) drug release profile desired, (d) surface charge and hydrophobicity of nanoparticles, (e) biocompatibility and biodegradability of nanomaterials, and (f) antigenicity and toxicity of the product [9]. Biopolymer-based nanoparticles including protein nanoparticles have gained considerable interest in recent years due to their many desirable properties such as low toxicity and biodegradability [10]. They are actively being developed for both pharmaceutical and nutraceutical delivery.

Proteins are a class of natural molecules that have unique functionalities and potential applications in both biomedical and material sciences [11]. They are deemed as ideal materials for nanoparticle preparation because of their amphiphilicity which allows them to interact well with both the drug and solvent [12]. Nanoparticles derived from natural proteins are biodegradable, metabolizable, and are easily amenable to surface modifications to allow attachment of drugs and targeting ligands [13]. They have been successfully synthesized from various proteins [13, 14] including water-soluble proteins (e.g., bovine and human serum albumin) and insoluble proteins (e.g., zein and gliadin). So far, there have been very few review articles on protein nanoparticles and most of them are focused on the preparation and characterization of nanoparticles derived from gelatin, albumin, and gliadin. In this review, we will discuss on a wide variety of proteins that have been used for protein nanoparticle formulations including the daily consumed soy and milk proteins, which have recently been reported, their methods of preparation, and their medical use with a focus on their application for cancer therapy.

## 2. Albumin

Albumin is a protein that can be obtained from a variety of sources, including egg white (ovalbumin), bovine serum albumin (BSA), and human serum albumin (HSA). Albumin is a major soluble protein of the circulating system and involved in the maintenance of osmotic pressure and binding and transport of nutrients to the cells. Many drugs and endogenous molecules are known to bind to albumin. Albumin serves as a depot and transporter protein [15]. This protein is freely soluble in water and diluted salt solution. The high solubility of albumin (up to 40% w/v) at pH 7.4 makes it an attractive macromolecular carrier capable of accommodating a wide variety of drugs. It is stable in the pH range of 4 to 9 and can be heated at 60°C up to 10 hours without any deleterious effects [16]. Albumin is widely used in the preparation of nanospheres and nanocapsules [17]. These albumin nanocarriers are biodegradable, easy to prepare, and have well-defined sizes and reactive functional groups (thiol, amino, and carboxyl) on their surface that can be used for ligand binding and other surface modifications. Drug release from albumin nanoparticles can be achieved naturally by protease digestion.

## 3. Gelatin

Gelatin is one of the proteinaceous materials that can be used for the production of nanoparticles. It is one of the most widely used animal proteins obtained by controlled hydrolysis of collagen, which is a major component of the skin, bones, and connective tissues [4]. Two different types of gelatin, A and B, can be produced following either acid or base hydrolysis, resulting in proteins with different isoelectric point (pI), molecular weight, amino acid composition, and viscosity [18]. For example, gelatin type A has the pI of 7–9, while gelatin type B has the pI of 4-5. Gelatin is generally regarded as a safe (GRAS) excipient approved by the United States FDA for use in pharmaceutical preparations such as gelatin capsules [19]. As pharmaceuticals, gelatin has long been considered as a biodegradable material since the early days of drug product development [12]. It is nontoxic and easy to be cross-linked or modified chemically. Therefore, it has an enormous potential to be used for the preparation of drug delivery systems such as microspheres and nanoparticles [20–22]. Gelatin has several other advantages including the following: (a) it is inexpensive, (b) it can be sterilized and nonpyrogenic, and (c) it possesses low antigenicity [23]. A key characteristic of gelatin is its high content of amino acids glycine, proline (mainly as hydroxyproline), and alanine. Most gelatin molecules contain repeating sequences of glycine, proline, and alanine triplets, which are responsible for the triple helical structure of gelatin [24]. Gelatin has many ionizable groups such as carboxyl, amino, phenol, guanidine, and imidazole, which are potential sites for conjugation or chemical modifications. Addition of chemical crosslinking agents such as glutaraldehyde gives gelatin stability, shape, and increased circulation time* in vivo *as compared to unmodified gelatin. The release of drugs from gelatin nanoparticles is dependent on the degree of crosslinking [20, 25]. Such crosslinking improves the integrity and performance of gelatin such as insolubility at high temperatures and reduced swelling in water [26]. Noncovalent crosslinking can be achieved through electrovalent and coordinate interactions [27]. These properties make gelatin-based nanoparticles a promising carrier system for drug delivery.

## 4. Elastin

Elastin is an essential component in connective tissues that is elastic and allows many tissues in the body to resume their shape after stretching or contracting [28]. Elastin is formed through lysine-mediated crosslinking of its soluble precursor tropoelastin. Tropoelastin is a 60–70 kDa protein whose length is dependent on its alternate splicing. Tropoelastin exists as a monomer in solution in two forms: an open globular molecule and a distended polypeptide [29]. The two types of elastin-derived polypeptides that have been used for drug delivery applications are *α*-elastin and elastin-like polypeptides (ELPs). *α*-Elastin, one of the soluble elastin-related polypeptides, has a unique feature that it undergoes aggregation under a selected condition of concentration and temperature called cloud point (CP). When the solution temperature is raised above the CP, *α*-elastin starts the complex self-assembly process that leads to aggregation. ELPs are repetitive peptide polymers with the sequence (Val-Pro-Gly-Xaa-Gly)_*n*_, where Xaa is a guest residue and *n* is the number of repetitive units [30]. These polypeptides are derived from tropoelastin and undergo an inverse phase transition which can be used to promote temperature-dependent self-assembly [31]. Below a tunable transition temperature (Tt), these ELPs are highly soluble. Above Tt, they coacervate into a secondary aqueous phase. This phase separation can be used to purify ELPs and their fusion proteins by a process called inverse transition cycling (ITC).

## 5. Gliadin and Legumin

Gliadin is a gluten protein found in wheat that exhibits bioadhesive property and has been explored for oral and topical drug delivery applications [32]. Gliadin is an attractive polymer for the preparation of mucoadhesive nanoparticles capable of adhering to mucus membranes. It has been used as a nanoparticle material because of its biodegradability, biocompatibility, and natural origin. Its hydrophobicity and solubility permit the design of nanoparticles capable of protecting the loaded drugs and controlling their release [14]. Gliadin nanoparticles exhibit a great tropism for upper gastrointestinal regions [33]. Its high capacity to interact with mucosa may be explained by its composition. This protein is rich in neutral and lipophilic amino acid residues. The neutral amino acids can promote hydrogen bonding with the mucosa, while the lipophilic residues can interact with biological tissues via hydrophobic interactions. Furthermore, gliadin contains amine and disulphide groups that are capable of developing bonds with mucin.

Legumin is one of the main storage proteins in pea seeds (*Pisum sativum *L.). It is an albuminous substance that resembles casein and functions as a source of sulfur-containing amino acids in seed meals. This protein can undergo self-assembly to form nanoparticles after aggregation or chemical crosslinking with glutaraldehyde [34].

## 6. Zein

Zein is a prolamine-rich protein that contains a high proportion of hydrophobic amino acids, proline, and glutamine [35]. It is a protein found in proteinaceous bodies from the endosperm of corn kernel. This hydrophobic protein is widely used for films and coatings. Zein is a GRAS polymer approved by the FDA for human applications. It has been used to prepare particulate systems for drug delivery and food applications [36]. Several studies utilized zein to produce edible capsules and films [37, 38]. Nanoparticles from zein proteins have been prepared to encapsulate several drugs and bioactive compounds including ivermectin, coumarin, and 5-fluorouracil (5-FU).* In vitro* release of coumarin was reported over 9 days from zein nanoparticles [36]. These studies demonstrated the utility of zein as a viable drug delivery material.

## 7. Soy Proteins

Soybean (*Glycine max* L.) is currently one of the most abundant sources of plant proteins. The enriched form of soy protein, known as soy protein isolate (SPI), has been reported to have high nutritional values and ingredient functionalities. A wide range of applications of soy proteins as food ingredients have been well documented [39]. In addition, SPI possesses a balanced composition of polar, nonpolar, and charged amino acids, allowing a variety of drugs to be incorporated. The major components of SPI are glycinin (MW = 360,000, ~60%) and *β*-conglycinin (MW = 180,000, ~40%) [40]. In an aqueous environment, these components exist as globular structures consisting of a hydrophilic shell and hydrophobic kernel, together with a certain amount of small water-soluble aggregates [41]. Upon addition of dissolvent or crosslinking agents, SPI molecules continue to aggregate and form various structures such as microspheres, hydrogels and polymer blends [42, 43]. Soy protein nanoparticles can be prepared either from a freshly prepared SPI by desolvation or from the glycinin fraction of defatted soy flour extraction using a simple coacervation method [43].

## 8. Milk Proteins

Milk contains several proteins with unique and diversified functional properties. The use of milk proteins as drug delivery vehicles is a new trend that has received much attention [44]. Two milk proteins that have been investigated for drug delivery applications are *β*-lactoglobulin (BLG) and casein. BLG is an 18.3 kDa protein containing two disulphide bonds and one free thiol group. The ability to preserve its native stable conformation at acidic pH makes it resistant to peptic and chymotryptic digestion [45]. BLG has a good gelling property which is useful in some drug delivery applications. Due to its abundance and low cost, BLG is a promising natural polymer for drug delivery applications [46]. Another potential milk protein for drug delivery applications is casein which exists as micelles in the size range of 100 to 200 nm [47]. Casein micelles can be regarded as a natural nanovehicle that delivers calcium and amino acids from mothers to offspring. Casein micelles have no fixed structures, and any changes in temperature, pH, ionic strength, water activity, and hydrostatic pressure will alter their size distribution because of their lack of rigid three-dimensional structure [48, 49]. Caseins have two distinct hydrophilic and hydrophobic domains that favor conformational changes in solutions depending on environmental conditions. Casein micelles contain small aggregates of 10 to 100 casein molecules that are held together by hydrophobic interactions and through calcium phosphate nanoclusters in the core. Their surface is covered by *κ*-casein which results in a charged surface that stabilizes the casein micelles by electrostatic and steric repulsions [50]. Casein micelles can withstand most processing treatments such as heat and mechanical forces [51].

## 9. Whey Proteins

Whey proteins are a mixture of globular proteins of variable composition and functional properties. Several whey protein products such as whey protein concentrates (WPC) and whey protein isolates (WPI) are industrially produced as food protein ingredients. The functional properties of these products are largely controlled by the major whey protein BLG. The whey protein and BLG preparations have been used as a vehicle for drug delivery. The use of whey proteins and specifically BLG as a drug delivery carrier is based mainly on the entrapment of these molecules in whey protein hydrogels. Hydrogels are water-swollen network of polymer that can hold a large amount of water while maintaining a network structure [52]. BLG is a suitable candidate for the preparation of drug delivery systems for lipophilic compounds because of its ability to bind hydrophobic constituents. Native BLG is stable in acidic conditions and is resistant to digestion by gastric proteases [53].

## 10. Methods of Preparation of Protein Nanoparticles

Preparation of protein nanoparticles is based on balancing the attractive and repulsive forces in the protein. It is generally accepted that increasing protein unfolding and decreasing intramolecular hydrophobic interactions are crucial to the formation of protein nanoparticles [46]. During such particle formation, the protein undergoes conformational changes depending on its composition, concentration, crosslinking, and preparation conditions such as pH, ionic strength, and type of solvent. Usually, surfactants are required to stabilize the nanoparticles of water-insoluble proteins such as gliadin [54]. Unfolding of proteins during the preparation process exposes interactive groups such as disulfides and thiols. Subsequent thermal or chemical crosslinking leads to the formation of cross-linked nanoparticles with entrapped drug molecules. Coacervation/desolvation and emulsion-based methods are most commonly used for the preparation of protein nanoparticles.

### 10.1. Coacervation/Desolvation

**Figure 1:**
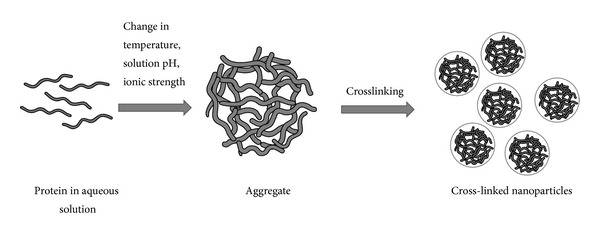
Preparation of protein nanoparticles by coacervation or phase separation method.

Coacervation or desolvation is based on the differential solubility of proteins in solvents as a function of solvent polarity, pH, ionic strength, and presence of electrolytes. The coacervation process reduces the solubility of the protein leading to phase separation (Figure 1). The addition of desolvating agent leads to conformation changes in protein structure resulting in coacervation or precipitation of the protein. By controlling processing variables, the size of nanoparticles in the coacervate can be controlled. After nanoparticles are formed, they are cross-linked by agents such as glutaraldehyde and glyoxal [55]. Organic solvents such as acetone and ethanol have been used as antisolvents for the preparation of protein nanoparticles. Thus far, coacervation/desolvation is the most commonly used method of preparation for protein nanoparticles. The effects of several factors on the formation of nanoparticles have been studied, especially with albumin nanoparticles. It was found that acetone when used as an antisolvent produces smaller albumin nanoparticles than those obtained by using ethanol [56]. An increase in antisolvent/solvent ratio decreases the particle size due to rapid extraction or diffusion of the solvent into the antisolvent phase, which limits the growth of particles [56]. Langer et al. [55] studied processing parameters that influence the formation of HSA nanoparticles. It was found that the pH prior to the desolvation step is a critical factor determining the size of nanoparticles. Higher pH values produce smaller nanoparticles with the size ranging from 100 to 300 nm. In this regard, it is essential to keep the pH away from the pI of protein to promote protein deaggregation and thus smaller nanoparticles [46]. High salt concentration can neutralize surface charges of the particles and promote agglomeration [55]. For BSA, increasing the protein concentration decreases the size of particles formed because of their increased nucleation upon antisolvent addition [56]. In the case of gelatin, the nanoparticles can be prepared by dissolving gelatin in an aqueous solution (pH 7), followed by changing the solvent composition from water to 75% v/v hydroalcoholic solution and upon gradual addition of ethanol with stirring [57]. In contrast, legumin is more hydrophobic and an increase in ionic strength of the solvent increases the protein solubility, thus producing smaller nanoparticles [58]. The effect of protein hydrophobicity on particle size was further studied in BLG and BSA nanoparticles. BLG which has a similar pI but lower hydrophobicity produces smaller nanoparticles (~130 nm) than BSA [46]. Denaturation of BLG by heat treatment prior to phase separation further reduces the particle size of BLG nanoparticles to approximately 60 nm. Orecchioni et al. studied gliadin nanoparticle formation using various ethanol/water ratios [59]. Smaller nanoparticles were obtained at the ethanol/water ratio that matches the solubility of gliadin and when the protein is in an expanded state.

Protein nanoparticles can be rigidized by crosslinking. An increase in the degree of crosslinking generally decreases the particle size due to the formation of denser particles [60]. Lysine residues in the protein are generally involved in the crosslinking. In the case of albumin, noncross-linked albumin nanoparticles coalesce to form a separate phase [55]. Therefore, crosslinking stabilizes the protein nanoparticles and reduces enzymatic degradation and drug release from the nanoparticles [60, 61]. However, it is essential to remove the cross-linkers as completely as possible afterward because of their toxicity [62]. Furthermore, the cross-linkers can affect the stability of drugs, particularly protein drugs in the nanoparticles. Surface coating can be used to stabilize nanoparticles instead of crosslinking. For example, cationic polymers such as polylysine and polyethyleneimine have been used to coat BSA nanoparticles to improve their stability [56]. Nanoparticles prepared from hydrophobic proteins such as gliadin and legumin generally require surfactants to stabilize the nanoparticles [58]. Poloxamer has been used to improve the solubility of legumin in the aqueous phase and stabilize the nanoparticles during phase separation. An increase in the surfactant concentration increases the product yield without appreciably altering the particle size [58]. For elastin-derived nanoparticles, a special technique using gamma irradiation crosslinking has been reported [63]. In this method, *α*-elastin aggregates were generated by increasing the temperature and then irradiated with ^60^Co gamma rays.

Drugs can be loaded into particles by surface adsorption or by entrapping the drugs in the particles during the preparation process. In the case of HSA nanoparticles, a higher drug loading efficiency was reported using the entrapment method as compared to surface adsorption method [64]. However, the loading efficiency depends on drug properties as well as other factors such as the drug/polymer ratio.

### 10.2. Emulsion/Solvent Extraction

**Figure 2:**
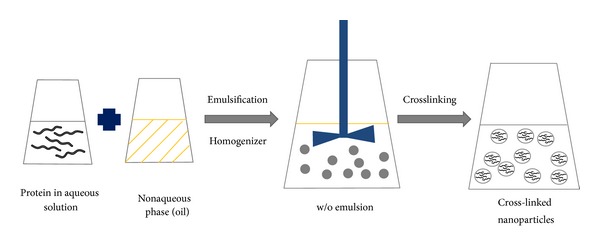
Preparation of protein nanoparticles by emulsion/solvent extraction method.

In this method, an aqueous solution of the protein is emulsified in oil by using a high-speed homogenizer or ultrasonic shear and the nanoparticles are formed at the w/o interface (Figure 2). Surfactants such as phosphatidylcholine and Span 80 are added as stabilizers to produce nanoparticles [65]. The oil phase is then removed using an organic solvent, thus forming nanoscopic proteinaceous particles where the size of the internal phase determines the ultimate size of particles. The emulsion-based method has been used to prepare a variety of protein nanoparticles including albumin and whey protein nanoparticles. HSA nanoparticles prepared by this method using olive oil as an oil phase have been reported [66]. Olive oil was slowly added to the aqueous protein solution containing phosphatidylcholine as a surfactant under constant mechanical stirring, followed by ultrasonication. The cross-linker glutaraldehyde was then added to the emulsion to obtain nanoparticles in the size range of 100 to 800 nm. The protein concentration and phase volume ratio (w/o) influence the particle size. Increasing the protein concentration and phase volume ratio enlarges the size of nanoparticles.

Thermal crosslinking can be used to replace chemical crosslinking. For example, Yang et al. prepared drug-loaded BSA nanoparticles using the emulsion method with thermal crosslinking [65]. In this method, an aqueous protein solution was emulsified with castor oil using Span 80 as a surfactant. The resulting emulsion was then added dropwise to heated (120–140°C) castor oil with constant stirring to evaporate the aqueous phase. This method of nanoparticle preparation using thermal crosslinking has also been used to prepare whey protein nanoparticles [67]. By mixing an aqueous solution of WPI with a mixture of oil and surfactant (limonene/n-butanol/Tween 60), a microemulsion was formed. This emulsion was then heated to 90°C for 20 minutes and immediately cooled and centrifuged. The supernatant was discarded and the pellet containing thermally aggregated whey proteins was washed thoroughly with ethanol. The formed nanoparticles were then heat-stabilized to obtain the final nanoparticle product. The size of nanoparticles prepared by the emulsion method is influenced by protein concentration and emulsification efficiency. In general, the size of particles prepared by the emulsion method is larger than that prepared by the coacervation method [68]. In either case, removal of the oil phase and organic solvent from the final products is essential for the safe use of the products.

### 10.3. Complex Coacervation

**Figure 3:**
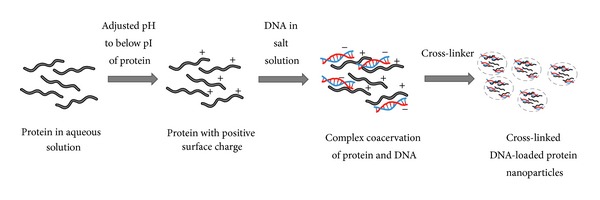
Preparation of protein nanoparticles by complex coacervation method.

This method of nanoparticle preparation is ideally suited for DNA entrapment, that is, for gene therapy applications. Since proteins are amphoteric with a large number of charged functional groups, they can be made cationic or anionic by adjusting the pH below or above the pI of the protein, respectively. The charged protein can then undergo electrostatic interactions with other polyelectrolytes (Figure 3) to facilitate the entrapment of DNA or oligonucleotides in the nanoparticles by coacervation. Salt-induced complex coacervation has been used to entrap DNA in gelatin nanoparticles [69]. At pH 5, gelatin is positively charged and can form complex coacervate with DNA. Salts such as sodium sulfate can be used to induce desolvation of the polyelectrolyte complex forming nanoparticles that can subsequently be stabilized by crosslinking agents. During the coacervation process, DNA is physically entrapped in the protein matrix. Endolysomotropic agents and other drugs can also be coencapsulated during the complex coacervation. Rhaese et al. prepared HSA-polyethyleneimine- (PEI-) DNA nanoparticles by inducing complex coacervation through charge neutralization [70]. The HSA solution (pH 4) was mixed with PEI and desolvation was achieved by adding sodium sulfate solution containing DNA. The nanoparticles were stabilized using the chemical cross-linker 1-ethyl-3[3-dimethylaminopropyl] carbodiimide (EDC), yielding nanoparticles in the size range of 300 to 700 nm. The ratio of HSA, PEI, and DNA plays a key role in determining the size of nanoparticles and their efficiency of gene transfection. Smaller nanoparticles (30–300 nm) can be prepared using a combination of HSA, DNA, and protamine [71]. Alternatively, cationized proteins can be used to form complex coacervates with DNA. Zwiorek et al. prepared a cationized gelatin by covalent attachment of cholamine (a quaternary amine) to the free carboxyl groups of gelatin using EDC as a coupling agent [72]. In the first step, gelatin nanoparticles were prepared by coacervation using acetone as a desolvating agent, which was followed by crosslinking with glutaraldehyde. Cholamine was then conjugated to the surface of gelatin nanoparticles at pH 4.5, and the resulting cationized gelatin nanoparticles were used to adsorb DNA at pH 7.4. These nanoparticles exhibited a neutral or slightly positive zeta potential with the size ranging from 183 to 288 nm. The cationized gelatin nanoparticles can also be formed by salt-induced complex coacervation with DNA as described above [73].

### 10.4. Electrospray

Electrospray is a relatively new technique for the preparation of protein nanoparticles. It has been used largely for the preparation of gliadin and elastin-like peptide nanoparticles [74, 75]. In this method, high voltage is applied to the protein solution supplied through an emitter which emits a liquid jet stream through a nozzle forming aerosolized liquid droplets. The aerosolized droplets contain protein nanoparticles of colloidal size which are collected. Drugs and nucleic acids can be easily incorporated into the nanoparticles with high efficacy using this method.

## 11. Protein Nanoparticle Technology and Cancer Therapy

Protein nanoparticles have been most extensively used for the delivery of anticancer drugs. Cancer is a major cause of death with no effective treatments. Approximately 12.7 million people were diagnosed with cancer worldwide in 2008 and this number is expected to increase to 21 million by 2030 [76]. Major methods of cancer treatment include surgery, radiation, chemotherapy, and immunotherapy. Each of these treatment modalities has advantages and disadvantages, and a combination of them is usually needed to produce the most effective results. Because most human cancers (>85%) are solid tumors, current cancer treatment strategies usually involve intrusive processes including the application of catheters for chemotherapy to shrink the tumors prior to their removal by surgery. This is then followed by more chemotherapy and/or radiation to kill the remaining tumor cells. Research efforts to improve the effectiveness of cancer therapy over the past 25 years have led to a substantial improvement in patient survival. However, problems associated with toxic side effects and poor quality of life in cancer patients remain a major issue [77].

Various drug delivery carriers have been used to improve the efficacy and reduce side effects of cancer therapy. Among these carriers, small biodegradable and biocompatible nanoparticles (<100 nm) have received the most attention. For systemic delivery of anticancer drugs, it is generally accepted that small particles (<500 nm) can avoid the reticuloendothelial system (RES), resulting in a longer circulation time [78]. In the case of solid tumors, small nanoparticles can extravasate through the leaky tumor vasculature, whereas they are excluded from intact vessels in normal tissues. It has been estimated that the pore size of tumor vasculature varies from 200 to 600 nm, and this has been exploited for passive targeting of nanoparticles to tumors [79]. During the last few decades, a large number of nanoparticle drug delivery systems have been developed for cancer therapy. Many liposomal systems, polymer-drug conjugates, and micellar formulations are part of the current state of the art in the clinics. An even greater number of nanoparticle platforms are currently in the various preclinical stages of development. Many of these delivery systems incorporate multifunctional and targeting capabilities in an effort to increase the efficacy of the delivery systems to combat the most difficult cancer challenges, including drug resistance and metastasis [80].

Ideally, for anticancer agents to be effective in cancer treatment, they should first be able to reach the target tumor tissues after administration by penetrating through various barriers in the body with minimal loss of content or activity in the blood circulation. Secondly, after reaching the tumor sites, they should have the ability to selectively kill tumor cells without adversely affecting normal cells. Thirdly, they should be released in a controlled manner in order to have the desired therapeutic effect. Through particle size and surface modifications, nanoparticles seem to have the potential to satisfy these requirements as effective drug carriers for cancer treatment. Protein-based nanoparticles are particularly interesting because they are relatively safe and easy to prepare, and their size distribution can be easily monitored [81]. They are also amendable to various modifications to incorporate functional and targeting capabilities. A protein-based nanocarrier system that has made an impact in cancer therapy is the albumin-bound nanocarrier system (~130 nm). A number of studies have shown that albumin accumulates in solid tumors [82] making it a potential carrier for targeted delivery of antitumor drugs. The approval of albumin-bound paclitaxel (Abraxane, ABI-008) by FDA for metastatic breast cancer exemplifies the clinical feasibility of this approach. Furthermore, several clinical trials currently in progress are using the albumin-bound nanocarrier system [83]. The system is prepared by mixing the drug (e.g., paclitaxel) with HSA in an aqueous solution, and the mixture passed through a high-pressure homogenizer to form drug-loaded albumin nanoparticles (100–200 nm). The use of HSA is based on the fact that albumin serves as a carrier for various endogenous and exogenous substances in the body [16]. Since albumin is a natural biological transporter of molecules across endothelial membranes via caveolae-mediated transcytosis, it is believed that albumin nanoparticles are taken up by cells via the caveolae pathway [84].

Abraxane is an albumin-bound paclitaxel formulation that has been shown to be superior to conventional paclitaxel formulations in various clinical trials [84]. Preclinical studies have shown that the concentration of paclitaxel bound to albumin in endothelial cells and extravascular space significantly increases (3–10 fold) [85, 86]. Data suggest that albumin may have an intrinsic targeting ability to tumors, although the enhanced permeability and retention (EPR) effect may play an additional role in the tumor accumulation. Overall, the albumin-bound paclitaxel formulation allows higher dosing than the standard paclitaxel (Taxol) formulation (260 mg·m^−2^ versus 175 mg·m^−2^, resp.) [86]. It also obviates the need for premedication with antihistamines and corticosteroids as is the case for Cremophor EL (paclitaxel in polyethoxylated castor oil). Most importantly, the patients can tolerate a higher paclitaxel dose with albumin-bound paclitaxel. Furthermore, the patients show a higher response rate and longer time to tumor progression without increasing the toxicity as compared to Cremophor EL formulation [84]. Abraxane is currently being tested as a first-line therapy in combination with other drugs (e.g., rapamycin and vorinostat) for metastatic breast cancer and other forms of cancer that have been shown to be sensitive to taxane drugs (e.g., ovarian and prostate cancers). Albumin is now being tested as a delivery platform for other drugs that have low water solubility such as rapamycin (~2.5 mg·mL^−1^). Albumin-bound rapamycin (ABI-009) has been in a clinical trial for the treatment of nonhematologic malignancies since 2008.

Cationic bovine serum albumin (CBSA) has recently been investigated as a novel siRNA delivery system for the treatment of metastatic lung cancer [87]. The preparation of cationic serum albumin is simple and the modification with its cationic group allows control of the protein's pI and surface charge for optimized drug delivery. Such modification also allows more efficient and targeted delivery of siRNA without increasing the toxicity during systemic applications. The CBSA can form stable nanosized particles with siRNA and protect the siRNA from degradation. CBSA also promotes the intracellular delivery of siRNA and its accumulation in the lung. When Bcl-2 siRNA is introduced into the systemic circulation using CBSA nanoparticles, it exhibits an efficient gene-silencing effect inducing cancer cell apoptosis and inhibiting tumor growth in a mouse model [87].

PEGylated nanoparticles have also been studied as a drug delivery carrier for cancer therapy. Surface modification of nanoparticles with polyethylene glycol (PEG) has been used to prepare long-circulating gelatin nanoparticles. PEGylated gelatin nanoparticles exhibit a twofold increase in plasma level as compared to normal gelatin nanoparticles. PEGylation also increases the accumulation of nanoparticles in tumors as demonstrated by a 6-fold increase in the half-life of PEGylated versus non-PEGylated nanoparticles in tumors [57]. Likewise, doxorubicin-loaded PEGylated nanoparticles are more efficient in inhibiting tumor growth than free doxorubicin or doxorubicin-loaded non-PEGylated nanoparticles [88].

Gliadin nanoparticles can be used as a bioadhesive delivery system for oral drug administration. The neutral amino acids in gliadin are believed to interact with the intestinal mucosa through hydrogen bonding, while the lipophilic amino acids in gliadin can interact with the mucus through hydrophobic interactions [32]. Such bioadhesion is thought to aid the sustained release delivery of anticancer drugs as well as colon cancer-targeted drug therapy. Gliadin nanoparticles have been used to carry anticancer drug cyclophosphamide. This nanodelivery system gradually releases the drug over a prolonged period of 48 hours and effectively induces apoptosis of breast cancer cells [74]. Zein nanoparticles are also a promising delivery system for anticancer drugs and diagnostic agents. There is a report of using zein nanoparticles containing 5-FU and quantum dot (QD) fluorophores for enhanced drug delivery and imaging of breast cancer [89]. These multifunctional QD nanoparticles are effective against breast cancer cells while providing a high quality imaging of the cancer [89].

Recently, several studies have demonstrated successful delivery of bioactive agents using milk protein nanoparticles. Zhen et al. prepared cisplatin-loaded casein nanoparticles and demonstrated their ability to penetrate cell membranes, target tumors, and inhibit tumor growth in hepatic tumor bearing mice [90]. Another study reported an effective anticancer activity of flutamide- (FLT-) loaded casein nanoparticles in prostate cancer bearing rats [91]. In the study, casein nanoparticles were prepared by emulsification at the pH below its pI and stabilized by crosslinking with sodium tripolyphosphate (TPP). The resulting nanoparticles were spherical and positively charged with the size of <100 nm. These particles slowly released FLT for up to 4 days and exhibited a higher antitumor activity than free FLT as judged by their ability to reduce tumor growth and PSA serum level [91]. Nanoparticles prepared from cow milk-derived lactoferrin have been evaluated as an oral delivery system for doxorubicin to treat hepatocellular carcinoma (HCC). The rationale behind this approach is that most metabolically active cancer cells including HCC cells express a high level of lactoferrin receptors and thus are a potential target of lactoferrin nanoparticles [92]. Doxorubicin-loaded lactoferrin nanoparticles were reported to exhibit improved efficacy, bioavailability, and safety as compared to free doxorubicin. This nanoparticle formulation was shown to reduce the number of liver nodules by >93% without affecting the body weight [93].

To promote drug targeting ability, protein nanoparticles have been chemically modified to incorporate targeting ligands that recognize specific cells and tissues. For example, Wartlick et al. modified albumin nanoparticles by covalently linking avidin to its surface, which was used to attach biotinylated HER2 antibody [94]. Such modification allows targeting of albumin nanoparticles to breast cancer cells which overexpress HER2. Another study reported the preparation of folic acid- (FA-) conjugated SPI nanoparticles. These nanoparticles exhibited smaller particle size, increased drug entrapment, and better cellular uptake than non-FA SPI nanoparticles [95], demonstrating the potential utility of this delivery system for cancer treatment.

## 12. Summary

The development of nanoparticle drug delivery systems is expected to have a major impact on the treatment of cancers and other life-threatening diseases. There is a great need to identify nanoparticle materials that are safe and effective in delivering therapeutic agents to the target sites. Protein polymers from natural sources are promising materials for constructing the nanocarrier systems. Of the various proteins for drug delivery applications, gelatin and albumin are most widely used, while plant proteins and milk proteins have just begun to be explored for drug delivery applications, and they represent highly promising protein nanomaterials. The commercial success of albumin-based nanoparticles has created a great interest in other proteins. By rationally designing protein nanoparticles based on their behaviors in the tumor microenvironment and based on cancer cell biology, improved efficacy and safety of cancer therapy can be achieved. In addition, multifunctional protein nanoparticles capable of carrying both therapeutic and diagnostic agents are now being explored for more effective cancer management. Although the application of protein nanoparticles for cancer therapy has already produced some exciting results and holds even greater promise in the future, comparison data on the performance and therapeutic efficiency of protein nanoparticles and other existing delivery systems are still lacking and represent a much needed area of research in the field.

## Figures and Tables

**Table 1:** Marketed nanosystems for cancer treatment.

Product name	Drug	Type of nanocarrier	Company
Daunoxome	Daunorubicin citrate	Liposome	Gilead Science, Cambridge, UK
Doxil	Doxorubicin HCl	Liposome	Johnson and Johnson, NJ, USA
Myocet	Doxorubicin	Liposome	Sopherion Therapeutics, NJ, USA
Caelyx	Doxorubicin HCl	Pegylated liposome	Johnson and Johnson, NJ, USA
Transdrug	Doxorubicin	Poly(alkylcyanoacrylate) nanoparticles	BioAlliance, Paris, France
Genexol-PM	Paclitaxel	Methoxy-PEG-polylactide nanoparticles	Samyang, South Korea
Oncaspar	Pegaspargase	PEG-asparaginase nanoparticles	Enzon, NJ, USA
Abraxane	Paclitaxel	Albumin-bound nanoparticles	American Bioscience, CA, USA

## Abbreviations

BLG: *β*-Lactoglobulin
BSA: Bovine serum albumin
CBSA: Cationic bovine serum albumin
CP: Cloud point
ELPs: Elastin-like polypeptides
FA: Folic acid
FDA: Food and Drug Administration
FLT: Flutamide
5-FU: 5-Fluorouracil
GRAS: Generally regarded as safe
HSA: Human serum albumin
PEG: Polyethylene glycol
PEI: Polyethyleneimine
pI: Isoelectric point
QD: Quantum dot
siRNA: Small interfering ribonucleic acid
SPI: Soy protein isolate
Tt: Transition temperature
WPC: Whey protein concentrates
WPI: Whey protein isolates.
